# Self-propulsion of Leidenfrost Drops between Non-Parallel Structures

**DOI:** 10.1038/s41598-017-12279-6

**Published:** 2017-09-20

**Authors:** Cheng Luo, Manjarik Mrinal, Xiang Wang

**Affiliations:** Department of Mechanical and Aerospace Engineering, University of Texas at Arlington, 500 W, First Street, Woolf Hall 226, Arlington, TX 76019 USA

## Abstract

In this work, we explored self-propulsion of a Leidenfrost drop between non-parallel structures. A theoretical model was first developed to determine conditions for liquid drops to start moving away from the corner of two non-parallel plates. These conditions were then simplified for the case of a Leidenfrost drop. Furthermore, ejection speeds and travel distances of Leidenfrost drops were derived using a scaling law. Subsequently, the theoretical models were validated by experiments. Finally, three new devices have been developed to manipulate Leidenfrost drops in different ways.

## Introduction

After a liquid drop is placed on a solid that is pre-heated well above the boiling point of the liquid, the drop levitates on a film of its own vapor, which is so-called Leidenfrost phenomenon^1–3^. A Leidenfrost drop has two specific properties^4^. First, due to lack of direct liquid/solid contact, this drop suffers almost no friction or adhesive force, enabling it to have high mobility. Second, it is in a non-wetting situation, and forms a contact angle of about 180° with its vapor-covered substrate.

At room temperature, a capillary force on a liquid drop may be created using an asymmetric structure, such as a conic capillary tube^5^, a conic fiber^6–10^, a pair of non-parallel plates^11–15^, or a spiral surface^16^. These structures have varied radii^5–10^, gaps^11–15^, or surface curvatures^16^ along their longitudinal directions. Accordingly, a gradient of Laplace pressure is produced inside a drop, resulting in a directional motion of the drop on such a structure.

In Leidenfrost regime, it is reported that ratchets, which consist of asymmetric tooth-like structures, e.g., saw-teeth and tilted pillars, enable directional movements of liquid drops^17–20^ or dry ice^21,22^. As commented in ref.^4^, one of main future directions in Leidenfrost research is to control ultramobile Leidenfrost drops, particularly when these drops are applied to remove heat from hot surfaces. For this purpose, in addition to ratchets, other asymmetric patterns should also be investigated^4^. Consequently, a question arises: how about those asymmetric structures that have already yielded self-propulsion of liquid drops at room temperature? That is, can they also be applied to control Leidenfrost drops? Due to non-wetting property of a Leidenfrost drop, conic fibers^6–10^ may not be a good option, since the Leidenfrost drop may not stick to their surfaces. The same applies to a spiral surface^16^. On the other hand, a liquid drop may be trapped inside a conic capillary tube^5^ or between two non-parallel plates^11–15^. Hence, the non-wetting property of a Leidenfrost drop is not a concern in these two cases. Nevertheless, conic tubes have 3-D holes, making it difficult to incorporate them on a solid surface, for example, to remove heat. Therefore, in this work, we focus on non-parallel plates.

Some feeding shorebirds, such as phalaropes, transport water drops to their mouths by squeezing and relaxing these drops with their long, thin beaks^11,23–26^. A beak may be visualized as two non-parallel plates^12^. This transporting process has been previously applied by us to develop a new artificial fog collector^13^. It is also found that, even if two non-parallel plates remain stationary, a liquid drop may still self-transport towards their corner^12,15^. According to criteria that we have previously derived^12^, this movement only occurs to a lyophilic drop. A lyophobic drop may run towards the open end of the two plates instead. However, it is unclear about what conditions should be satisfied to make this happen. It is important to know these conditions, as well as speeds and travel distances, to control Leidenfrost drops using the non-parallel plates. Consequently, the corresponding theory is explored here.

## Moving Conditions

**Figure 1:**
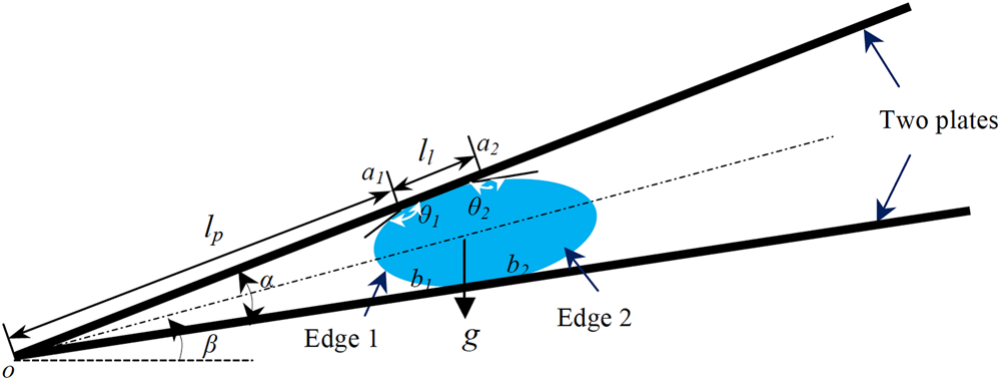
Cross-sectional schematic of a liquid drop placed between two tilted, non-parallel plates.

Following a line of reasoning used in refs^6,12,27^, we derive Laplace pressure to find moving conditions. Figure 1 shows cross-sectional schematic of a liquid drop squeezed between two non-parallel plates. For simplicity, the left and right edges of the liquid drop are called “Edge 1” and “Edge 2”, respectively. Use *o* and *α*, respectively, to denote apex edge and apex angle of the two plates. Let *a*_1_ and *b*_1_ denote the two points that Edge 1 intersects with the top and bottom plates, separately, and set *a*_2_ and *b*_2_ to be the two intersecting points that Edge 2 forms with these two plates. Use *l*_*p*_ to denote the distance between *o* and *a*_1_, and let *l*_*l*_ be the length of *a*_1_ *a*_2_. Set *β* to be the tilt degree of the middle plane between the two plates, and ${0}^{o}\le \beta \le {90}^{o}.$ Let *θ*_1_ represent equilibrium contact angle at *a*_1_ and *b*_1_, and use *θ*_2_ to stand for the one at *a*_2_ and *b*_2_. Set *θ*_*adv*_ and *θ*_*rec*_ to be, respectively, advancing and receding contact angles. Then, both *θ*_1_ and *θ*_2_ vary between *θ*_*adv*_ and *θ*_*rec*_. Let ${p}_{w1}$ and ${p}_{w2}$ denote the liquid pressures at Edges 1 and 2, respectively. Set *p*_*a*_ to be the pressure of surrounding air. Use *R*_1_ and *R*_2_ to represent the radii of Edges 1 and 2, separately. Meanwhile, let *R*_12_ and *R*_22_ stand for the radii of the curves on the drop surface, which are perpendicular to Edges 1 and 2, respectively. A radius is considered positive if its associated curve on the drop surface bends towards air. Subsequently, in terms of Young-Laplace equation^28^, we have

1 $${p}_{w1}=\gamma (\frac{1}{{R}_{1}}+\frac{1}{{R}_{12}})+{p}_{a},$$

2 $${p}_{w2}=\gamma (\frac{1}{{R}_{2}}+\frac{1}{{R}_{22}})+{p}_{a},$$

where *γ* denotes surface tension of the air/liquid interface. $({p}_{w1}-{p}_{a})$ and $({p}_{w2}-{p}_{a})$are so-called Laplace pressures.

Two assumptions are made for subsequent derivation. First, half thicknesses of liquid drops are less than capillary length of the corresponding liquid, which is 2.7 mm in the case of water at room temperature. Second, the plate gap is much smaller than the drop radius. According to the first assumption, across drop thickness, the gravity effect on Laplace pressure is neglected. Hence, ${p}_{w1}$ and ${p}_{w2}$ are uniform on Edges 1 and 2, respectively^6,14,15^. Subsequently, both Edges 1 and 2 are considered to be circular arcs^6^. It follows from geometric analysis that

3 $$\frac{1}{{R}_{1}}=\frac{-\,\cos (\frac{\alpha }{2}-{\theta }_{1})}{{l}_{p}\,\sin \,\frac{\alpha }{2}},$$

4 $$\frac{1}{{R}_{2}}=\frac{-\,\cos (\frac{\alpha }{2}+{\theta }_{2})}{({l}_{p}+{l}_{l})\sin \,\frac{\alpha }{2}}.$$

In the previous work^12^, we assumed that gravity effect was also negligible along the longitudinal direction of a drop. However, by observing Figs 3(b2),(c2) and 5(a2) of ref.^12^, when drop size is large or apex angle is small, such as 0.7°, half of *l*_*l*_ may be larger than capillary length of a liquid. Consequently, gravity effect is considered in this work along the longitudinal direction of a drop.

**Figure 2:**
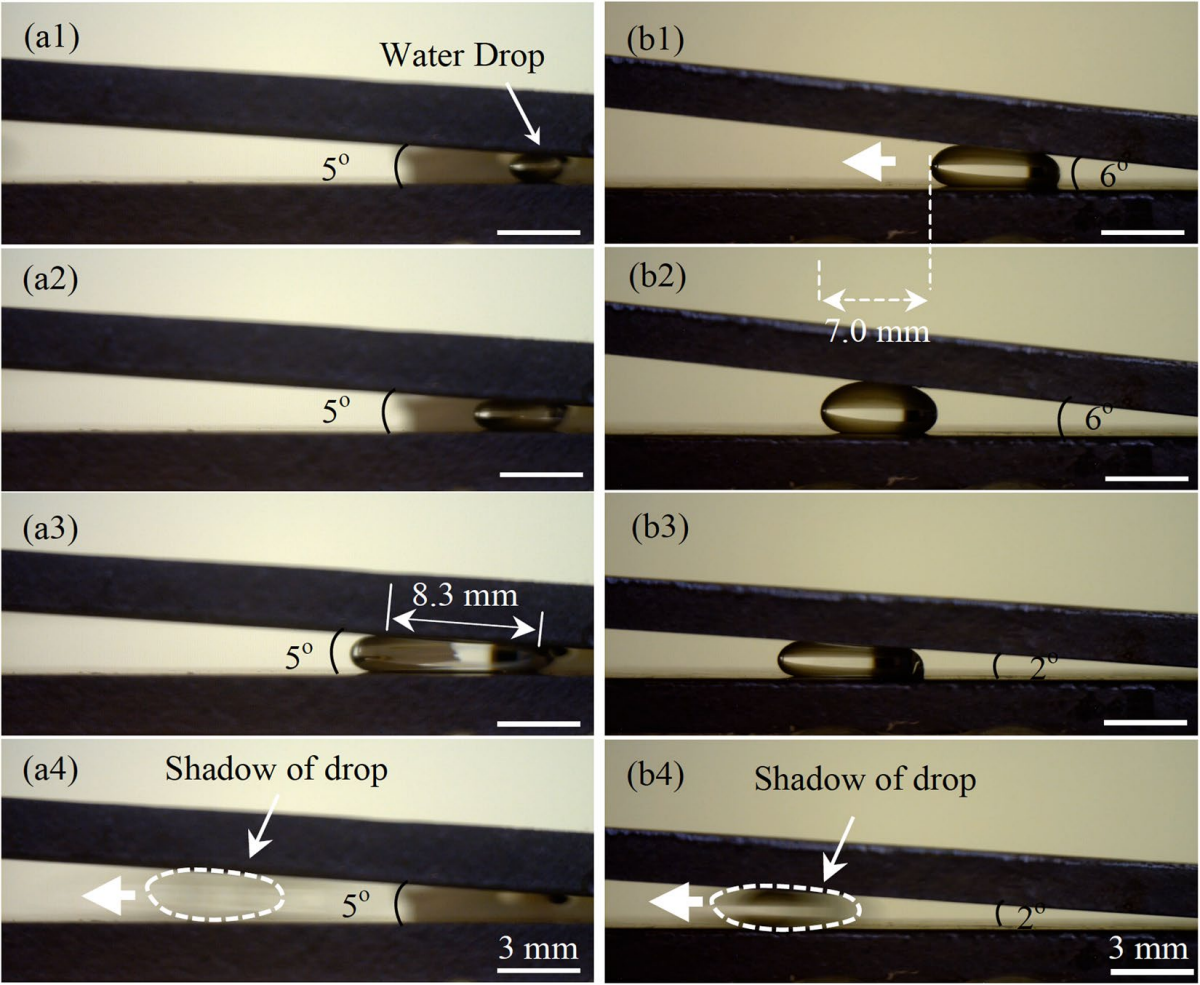
Two different approaches to drive a water drop away from the corner of two Glaco-covered plates. (**a1**–**a3**) For a fixed apex angle of 5°, when *l*_*l*_ was increased to 8.3 mm by supplying additional water, (**a4**) the water drop ran away from the corner with a speed of 6.9 cm/s. (**b1**–**b2**) For a drop with a fixed volume, as the apex angle was reduced to 6° by lowering down the top plate, the water drop moved a distance of 7.0 mm and stopped; and (**b3**–**b4**) further reducing the apex angle to 2° enabled the water drop to move with a speed of 3.7 cm/s. These two tests were both done at room temperature. Thick arrows in (**a4**), (**b1**), and (**b4**) represent moving directions of drops, and the drops in (**a4**) and (**b4**) are surrounded by dotted loops for clarity. The same applies to the following figures.

Since *R*_1_ and *R*_12_ are, respectively, in the same order as the plate gap and drop radius, according to the above second assumption, *R*_1_ is much smaller than *R*_12_, which has been previously validated by our experimental results in ref.^14^ (see its Fig. 2). Accordingly, in comparison with $\frac{1}{{R}_{1}}$, the effect of $\frac{1}{{R}_{12}}$ on ${p}_{w1}$ can be neglected. Likewise, during the consideration of ${p}_{w2}$, the effect of $\frac{1}{{R}_{22}}$ is also neglected. With the aid of (1)–(4), along the longitudinal direction of the drop, we have

5 $$\frac{{\rm{\Delta }}p}{{l}_{l}}=-\frac{\gamma \,\cos \,({\theta }_{1}-\frac{\alpha }{2})}{{l}_{l}({l}_{p}+{l}_{l})\sin \,\frac{\alpha }{2}}+\frac{\gamma \,\cos \,({\theta }_{2}+\frac{\alpha }{2})}{{l}_{l}{l}_{p}\,\sin \,\frac{\alpha }{2}},$$

where $${\rm{\Delta }}p={p}_{w1}-{p}_{w2}.$$

In the previous work^12^, the air/liquid interfaces between the two plates were assumed to have approximately cylindrical shapes, which implied that both $\frac{1}{{R}_{12}}$ and $\frac{1}{{R}_{22}}$ were zero. Based on the assumption, a 2-D model was developed in ref.^12^ to consider wetting phenomena. Since a squeezed drop has a pancake shape, this assumption is relaxed in the present work. Although a 3-D model is established here for the drop, due to our second assumption, $\frac{1}{{R}_{12}}$ and $\frac{1}{{R}_{22}}$ are still neglected in determining liquid pressures. As a result, the derived gradient of Laplace pressure, i.e., Eq. (5), is equivalent to what was obtained in ref.^12^ (see its Eqs (3) and (4)).

The gradient of Laplace pressure given in Eq. (5) is caused by surface tension. There also exists a gravity-induced pressure gradient along the longitudinal direction of the drop. It is $\rho g\,\sin \,\beta ,$ where *ρ* and *g*, respectively, denote mass density of the liquid and gravitational acceleration.

If

6 $$\frac{{\rm{\Delta }}p}{{l}_{l}} > \rho g\,\sin \,\beta ,$$

then the resultant gradient of liquid pressure is positive along the direction from Edges 1 towards 2. Accordingly, liquid flows along this direction inside the drop, which makes Edge 1 bend less toward air while Edge 2 more. Consequently, *θ*_1_ is reduced, but *θ*_2_ is increased. As observed from Eq. (5), $\frac{{\rm{\Delta }}p}{{l}_{l}}$ is decreased accordingly. In case $\frac{{\rm{\Delta }}p}{{l}_{l}}$ becomes balanced with $\rho g\,\sin \,\beta$, the drop remains stationary. During this process, the flow speed inside the drop is small, since Edges 1 and 2 are still pinned and just slightly change their shapes. Accordingly, inertial and viscous forces are negligible. If In eq. (6) still holds true when *θ*_2_ is increased to *θ*_*adv*_, then Edge 2 starts to move towards the open end of the two plates, followed by Edge 1. Subsequently, the liquid may have a high flow speed. Further consideration of its force balance should involve dynamic forces, such as inertial and viscous forces. Here, we focus on deriving conditions to judge whether a drop can start a motion. Thus, we only consider the balance of forces on a stationary drop, which does not include the dynamic forces. As will be seen later, in determining ejection speed and travel distance of a Leidenfrost drop, we estimate the effects of these dynamic forces.

As observed from Eq. (5), when a drop escapes from the plate corner, due to increase in *l*_*l*_, $\frac{{\rm{\Delta }}p}{{l}_{l}}$ gradually decreases. After the drop travels a certain distance, In eq. (6) may be violated. As a result of this violation, the drop may then stop. Further consideration of In eq. (6) results in the following criterion:

if

7 $${\theta }_{rec} > (\frac{\pi }{2}+\frac{\alpha }{2}),$$

8 $$[\frac{{\lambda }^{2}\,\cos \,({\theta }_{adv}+\frac{\alpha }{2})}{{l}_{l}({l}_{p}+{l}_{l})\sin \,\frac{\alpha }{2}}-\frac{{\lambda }^{2}\,\cos \,({\theta }_{rec}-\frac{\alpha }{2})}{{l}_{l}{l}_{p}\,\sin \,\frac{\alpha }{2}}] > \,\sin \,\beta ,$$

where *λ* stands for capillary length of a liquid and it equals ${(\frac{\gamma }{\rho g})}^{\frac{1}{2}}$, then a liquid drop that is put between two non-parallel plates should start moving towards the open end of these two plates.

This criterion is proved below. Let ${({p}_{w1}-{p}_{w2})}_{\min }$ represent the minimum value of the Laplace pressure difference for fixed *l*_*l*_, *l*_*p*_ and *α*. According to monotonically decreasing property of cosine functions, it follows from Eq. (5) that

9 $${({p}_{w1}-{p}_{w2})}_{{\rm{\min }}}=\frac{-\gamma \,\cos \,({\theta }_{rec}-\frac{\alpha }{2})}{{l}_{p}\,\sin \,\frac{\alpha }{2}}+\frac{\gamma \,\cos \,({\theta }_{adv}+\frac{\alpha }{2})}{({l}_{p}+{l}_{l})\,\sin \,\frac{\alpha }{2}},$$

which corresponds to the case that *θ*_1_ and *θ*_2_ equal *θ*_*rec*_ and *θ*_*adv*_ respectively. If In eqs (7) and (8) hold true, then, by In eq. (7), it can be shown that

10 $${({p}_{w1}-{p}_{w2}-\rho g\sin \beta )}_{{\rm{\min }}} > 0.$$

This inequality means that In eq. (6) holds true for any allowed *θ*_1_ and *θ*_2_. Consequently, due to the pressure gradient, the drop should escape from the plate corner. Thus, In eqs (7) and (8) form a sufficient condition for the liquid drop to run towards the open end of the two plates.

In the case of a Leidenfrost drop, ${\theta }_{rec}={\theta }_{adv}\approx {180}^{{\rm{o}}}.$ Hence, In eq. (7) is satisfied, and In eq. (8) is simplified as

11 $$\frac{{\lambda }^{2}}{\tan \,\frac{\alpha }{2}{l}_{l}({l}_{p}+{l}_{l})} > \,\sin \,\beta ,$$

which is the only condition that should be satisfied for a Leidenfrost drop to move away from the plate corner.

When the middle plane of the two plates is oriented horizontally, i.e., $\beta ={0}^{o}$, In eq. (11) is automatically met. This result implies that, given that the movement is along the horizontal direction, regardless of apex angle, drop size and drop location, a Leidenfrost drop is capable of continuously transporting towards the open end of the two plates.

A Leidenfrost drop has a directional movement on a ratchet, due to the propulsion of the ratchet’s reaction forces that are induced by self-rotation of the drop^29^. The driving mechanism is different here. As observed from our theoretical model, the motion is caused by uneven distribution of liquid pressure inside a squeezed drop. This self-propulsion may also be explained in terms of squeezing forces and contact angle hysteresis. The two non-parallel plates provide forces to squeeze a drop. These squeezing forces are perpendicular to the plate surfaces, and point towards the drop. Their resultant force points to the direction from the plate corner towards the open end of the two plates. Meanwhile, due to contact angle hysteresis, the drop also suffers a friction force. This force may balance with the aforementioned resultant force, and thus prevents the drop from moving towards the open end of the two plates. For a Leidenfrost drop, the friction force becomes zero, since contact angle hysteresis is zero. As a result, the drop only suffers the squeezing forces. These forces entrain this drop.

## Ejection speed and moving distance

We estimate ejection speed and travel distance of a Leidenfrost drop using a scaling law. During the ejection process, the squeezed drop becomes more spherical with a decreasing surface area. The squeezing forces are symmetric with respect to the middle plane of the drop. Hence, they do not rotate this drop, and only induce a translational movement. Accordingly, the part of surface energy that is lost due to reduction in surface area is converted to the drop's center-of-mass translational kinetic energy. The lost surface energy is related to initial contact area between the drop and the structures, which is on an order of 0.5$\pi {{l}_{l}}^{2}.$ Hence, it scales as 0.5*γ* $\pi {{l}_{l}}^{2}.$ Let *V* and *u*, respectively, represent volume of the drop and speed of the drop’s center of mass. Balancing the lost surface energy with the translational kinetic energy, 0.5*ρVu* ^2^, we have $u \sim \lambda {l}_{l}{(\frac{\pi g}{V})}^{\frac{1}{2}}.$ The volume of the drop may scale as $V \sim \frac{\pi t{{l}_{l}}^{2}}{4},$ where *t* denotes average thickness of the drop and it approximately equals gap size of the two plates at the drop center. Consequently, we have

12 $$u \sim 2\lambda {(\frac{g}{t})}^{\frac{1}{2}}.$$

This relation indicates that ejection speed of a Leidenfrost drop is inversely proportional to ${t}^{\frac{1}{2}}$.

Next, using Relation (12), we evaluate ejection speeds of water and Isopropyl alcohol (IPA) drops. In Leidenfrost states, the temperatures of water and IPA drops are close to their boiling points^4,30^, which are 100 °C and 82 °C, respectively. At such temperatures, the values of *γ* and *ρ* are, respectively, 58.9 mN/m and 960 kg/m^3^ for water^31,32^, and they are 17.0 mN/m and 728 kg/m^3^ for IPA^33^. Subsequently, the values of *λ* are 2.5 mm and 1.5 mm, respectively, for water and IPA. According to Relation (12), with *t*~1 mm and *g* = 9.8 m/s^2^, ejection speeds of both water and IPA drops have the order of 10 cm/s, which is in the same order as those reported on ratchets^17–19,29^. If *t* is an order higher than 1 mm, then, by Relation (12), the ejection speeds should be an order lower than 10 cm/s. In case *t*~10 µm, these speeds would be increased by one order of magnitude to 1 m/s. However, because this thickness is even smaller than that of the vapor film between a Leidenfrost drop and its substrate, the drop might have already completely evaporated before its ejection. Thus, in practice, to achieve a high ejection speed, non-parallel structures should have a mm-scaled gap.

Let *s* denote the distance that an ejected Leidenfrost drop is capable of travelling on a tilted substrate. In deriving Relation (12), gravity effect was neglected, which holds true if $\beta ={0}^{o}.$ Nevertheless, when $\beta  > {0}^{o}$, it should be counted. For this purpose, the location of the drop center, which corresponds to the moment that the drop just starts its ejection process, is defined as the starting point of *s*. With the aid of Relation (12), balancing the kinetic energy with gravitational potential energy, *ρVgs* $\sin \,\beta$, we have

13 $$s \sim \frac{2{\lambda }^{2}}{t\,\sin \,\beta },$$

where ${0}^{{\rm{o}}} < \beta \le {90}^{o}.$ It can be observed from this relation that *s* is inversely proportional to $\sin \,\beta$.

## Effects of dynamic and friction forces

In deriving Relations (12) and (13), we neglected effects of surface tension gradient, inertial force and viscous force inside a drop, as well as those of friction forces outside the drop. As what was done in ref.^4^, we estimate these effects.

A Leidenfrost drop may have self-rotation, which is a Marangoni thermocapillary flow^30–35^. The temperature of the drop’s bottom surface equals boiling point of the corresponding liquid. It is a few degrees higher than that of the drop’s top surface^30^. This temperature difference results in a gradient of surface tension, generating the Marangoni flow that circulates inside the drop^30,35^. For both water and IPA, the flow speed has an order of 10 cm/s^4,29^. Given that an ejected drop’s center of mass has a translational speed with this order as well, the resultant speed of a point in the drop, *u*_0_, should also have an order of 10 cm/s.

Weber (*We*), Capillary (*Ca*) and Marangoni (*Ma*) numbers represent relative magnitudes of inertial, viscous and Marangoni forces with that of surface tension force. We have $We=\frac{\rho {{u}_{0}}^{2}r}{\gamma }$, $Ca=\frac{\mu {u}_{0}}{\gamma }$, and $Ma=\frac{{\rm{\Delta }}\gamma }{\gamma }$, where *r* is equatorial radius of the drop, *μ* is dynamic viscosity of the liquid, and Δ*γ* is the difference in surface tension caused by the temperature variation between the bottom and top surfaces of the drop. Δ*γ* is in the order of 0.1 mN/m for both water^31^ and IPA^33^. The values of *μ* for water^32^ and IPA^33^ are about 0.6 and 0.3 mPa S, respectively, at temperatures close to their respective boiling points. Also, *r*~1 mm. In the case of either water or IPA, the corresponding Weber, Capillary and Marangoni numbers are found to be all less than unity, indicating that the surface tension force dominates the drop shape during the ejecting and transporting processes.

A drop suffers two main friction forces from surrounding environment: viscous friction in the vapor film, and inertial friction in the air. These two forces scale as $\frac{{\mu }_{v}{u}_{0}{r}^{2}}{h}$ and ${\rho }_{v}{{u}_{0}}^{2}{r}^{2}$, respectively, where *μ*_*v*_ and *ρ*_*v*_ are dynamic viscosity and mass density of vapor, and *h* is thickness of the vapor film^4^. For vapors of both water and IPA, we have *μ*_*v*_ $\approx$ 0.03 mPa S,*ρ*_*v*_ ~1 kg/m^3^ and *h* ≈ 150 µm^36^. In the case of either water or IPA, the ratios of the two friction forces with $\rho gV\,\sin \,{1}^{o}$ are less than 0.1. The latter is the gravitational force that a drop suffers along its moving direction when the substrate is slightly tilted by 1°. This result implies that, in comparison with gravity, the effects of the two main friction forces on the motions are negligible.

In summary, according to the above discussions, it is reasonable to neglect the aforementioned effects in deriving Relations (12) and (13).

## Experimental Validation

Four experiments were performed on water and IPA drops. In the first experiment, *β* was controlled to be approximately 0° to examine In eq. (8) at room temperature (around 25 °C). In the second and third experiments, Relations (12) and (13) were, respectively, evaluated using Leidenfrost drops. In the fourth experiment, the effect of substrate temperature gradient on moving direction was evaluated.

The volumes of tested drops ranged from 20 to 300 µL. An optical microscope (model: Dino-Lite Pro Digital Microscope) was employed in the first experiment to record drop behaviors between two non-parallel plates with a rate of 15 frames per second. A digital camera (model: Canon t1i) installed with EF-S 18-55mm f/3.5-5.6 IS Lens was chosen to record the other experiments with a rate of 30 frames per second.

In the first experiment, we spray-coated Al plates with Glaco (Glaco Mirror Coat Zero, Soft99 Co., Japan), which was a super-hydrophobic material at room temperature^37,38^. Since IPA etched Glaco, only water was tested. It had receding and advancing contact angles of 154° and 161°, respectively, on Glaco-coated plates, indicating that In eq. (7) was satisfied. When *β* = 0°, it follows from In eq. (8) that

14 $$\frac{{l}_{l}}{{l}_{p}} > [\frac{\cos \,({\theta }_{adv}+\frac{\alpha }{2})}{\cos \,({\theta }_{rec}-\frac{\alpha }{2})}-1].$$

As observed from this inequality, there were two approaches to make it satisfied. The first experiment included two sets of experiments. These two approaches were examined in the first set of tests. The first method was to increase drop size. As seen from Fig. 2(a), a small water drop was difficult to detach from a needle that was used to deliver the drop to the plates, since the Glaco coating was less wetting than the needle. However, when *l*_*l*_ was increased to 8.3 mm, the corresponding driving force was larger than pinning force of the needle, enabling the drop to get off from the needle and begin to move with a speed of 6.9 cm/s. The second approach was to decrease *α*. As observed from Fig. 2(b), when *α* was decreased to 6° by gradually lowering down the top plate using a micrometer, a water drop began to transport away from the plate corner. It stopped after travelling about 7.0 mm. If *α* was further decreased to 2°, then the drop started to move again with a speed of 3.7 cm/s. In addition, during the validation of both approaches, In eq. (8) was satisfied at the moment that a drop started to move, which was examined by substituting the corresponding experimental data into this inequality.

**Figure 3:**
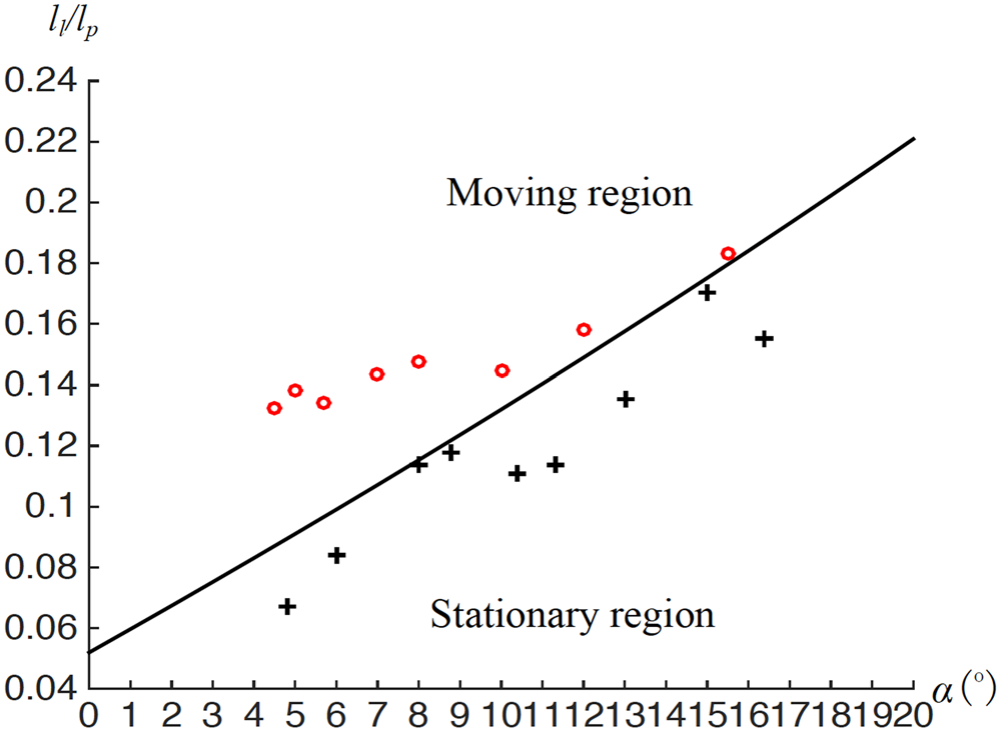
Moving conditions of drops for different combinations of *l*_*l*_/*l*_*p*_ and *α*. Moving and stationary regions were determined according to In eq. (14): the moving region satisfies In eq. (14), while the stationary does not. Data points represent experimental results. Red “o” indicates that a drop moved towards the open end of two plates, while black “+” means that a drop was stationary.

In the second set of tests, to avoid disturbance of a needle, drops were first released at different locations of the bottom plate, the top plate was then slowly lowered down to press them. Subsequently, the values of *α*, *l*_*l*_, and *l*_*p*_ were measured, and the corresponding data point (*α*, $\frac{{l}_{l}}{{l}_{p}}$) was plotted in Fig. 3. According to the derived criterion, when In eq. (14) is met, a liquid drop should move towards the open end of the plate. Otherwise, it should be stationary. As shown in Fig. 3, all the results in the second set of tests matched these theoretical predictions. In summary, given that *β* is approximately 0°, the derived criterion was validated by the first experiment at room temperature.

**Figure 4:**
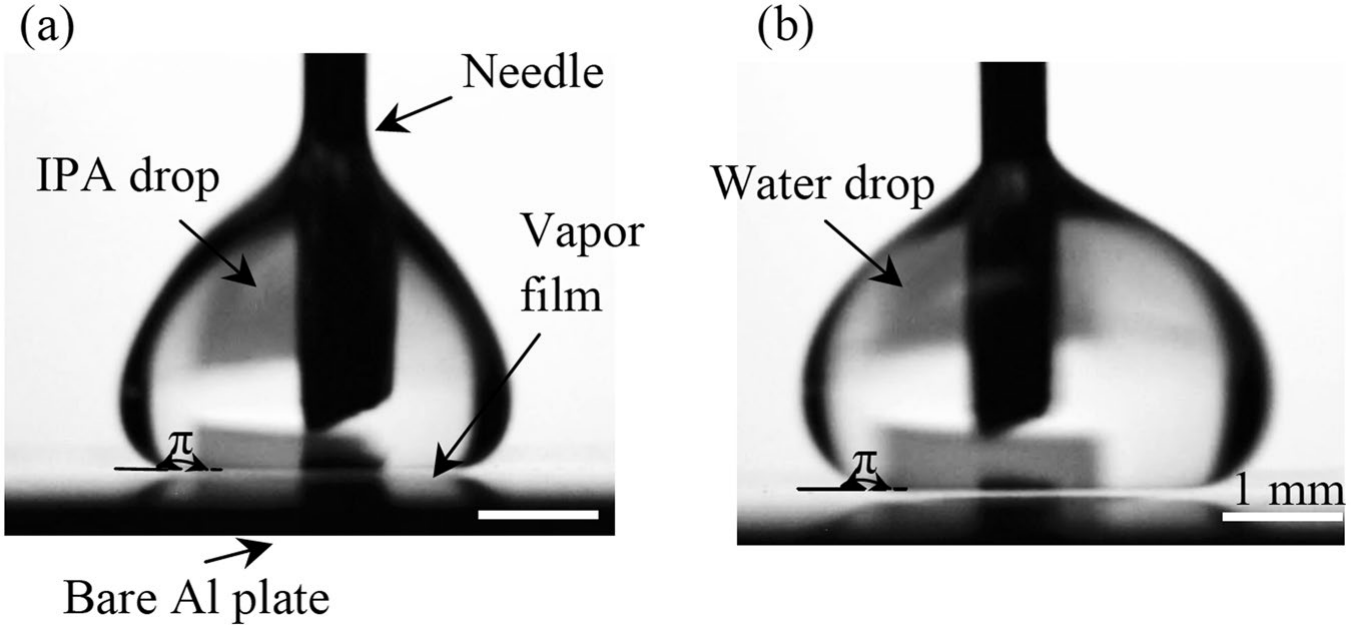
Representative images of (**a**) IPA and (**b**) water drops when underlying Al plates were heated to 115 °C and 215 °C, respectively. They were pinned on the Al plates using a needle to obtain clear images.

In the second experiment, *β* was still set to be 0°. Leidenfrost points of water and IPA were first measured on bare Al plates. They were 215 and 115 °C, respectively, with an error of 10 °C. This error was induced due to two factors: (i) the variation of temperature across an Al plate, and (ii) the hot plate that was used to heat the Al plates did not fix its surface temperature at the pre-set value. As shown in Fig. 4(a) and (b), at these temperatures, vapor films with thicknesses of 100 to 200 µm were clearly observed between the corresponding liquid drops and their substrates. Due to the existence of this vapor film, a Leidenfrost drop did not have direct contact with the plate, and it sat on the film instead. Also, the corresponding contact angles were about 180°.

In the subsequent tests of this work, unless otherwise stated, substrate temperatures were in the range of 215 to 250 °C for water, and they were between 115 and 125 °C for IPA. These substrate temperatures ensured that the corresponding drops were in Leidenfrost states. Due to high mobility, when a Leidenfrost drop was released on a plate, it moved around. Accordingly, the approach that was used in the first experiment for the second set of tests could not be applied to position a Leidenfrost drop. As such, a channel with non-parallel sidewalls was employed to replace the two plates. The channel was formed between two 4-mm-high, 10-cm-long Al bars, which were located on an Al plate. The apex angle of this channel could be varied by adjusting relative orientations of the two bars.

**Figure 5:**
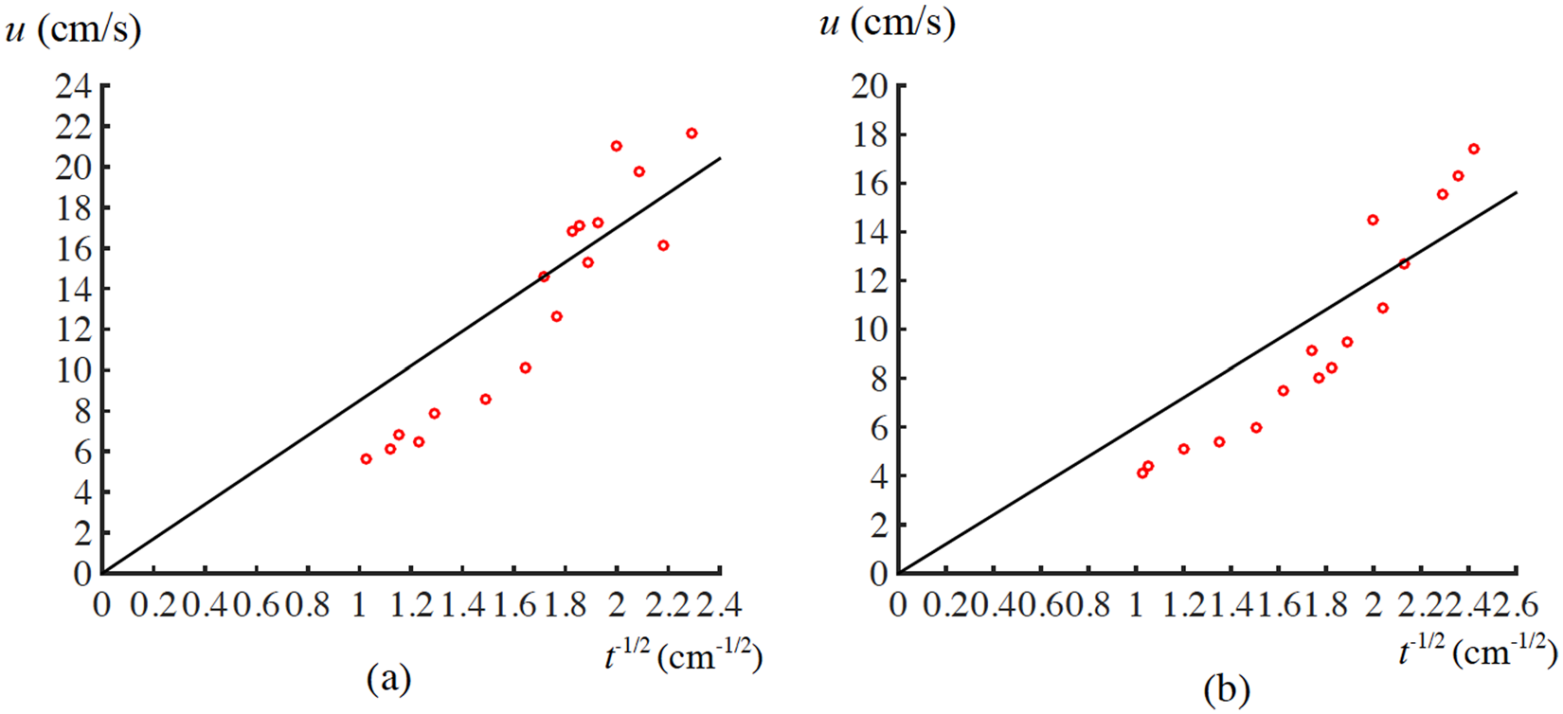
Relations of *u* with *t* ^−1/2^ for (**a**) water and (**b**) IPA drops. Lines and scattered points, respectively, represent fitted and experimental results.

The channel surface was lyophilic. When a released drop, which originally had room temperature, just contacted the channel, it was not in Leidenfrost state, and its contact angle with the channel surface was still less than 90°. Hence, although the released drop had a size larger than its underneath channel gap, it could get inside the channel^39^. Once a drop went into the channel and had contact with the two channel sidewalls, it began to move immediately. The drop continued to move till it got out of the channel. To validate Relation (12), a set of data points $({t}^{\frac{-1}{2}},u)$ was determined. For water, the values of *t* ranged from 1.9 to 9.5 mm, and the corresponding speeds decreased from 21.7 to 5.6 cm/s. In the case of IPA, the values of *t* varied from 1.7 to 9.5 mm, and the related speeds changed from 17.4 to 4.1 cm/s. Three points were observed from experimental results (Fig. 5). First, for either water or IPA, *u* approximately had a linear relation with ${t}^{\frac{-1}{2}}$. Second, both water and IPA drops had speeds with an order of 10 cm/s. Third, due to their larger *λ*, in general the water drops moved faster than the IPA. All the three points matched the corresponding theoretical predictions of Relation (12).

Relation (12) was derived for the case that a squeezed drop had a pancake shape. Due to the effect of a channel’s bottom surface, this relation is an approximate one in the case of a channel. A drop is flattened at its interface with the channel’s bottom surface. Thus, the drop does not have an ideal pancake shape. As such, we re-write Relation (12) as

15 $$u=2k\lambda {(\frac{g}{t})}^{\frac{1}{2}},$$

where *k* is a numerical factor for correcting our “pancake” assumption in the case of a channel. By curve-fitting Eq. (15) with experimental data, the values of *k* are found to be 0.54 and 0.64, respectively, for water and IPA (Fig. 5).

**Figure 6:**
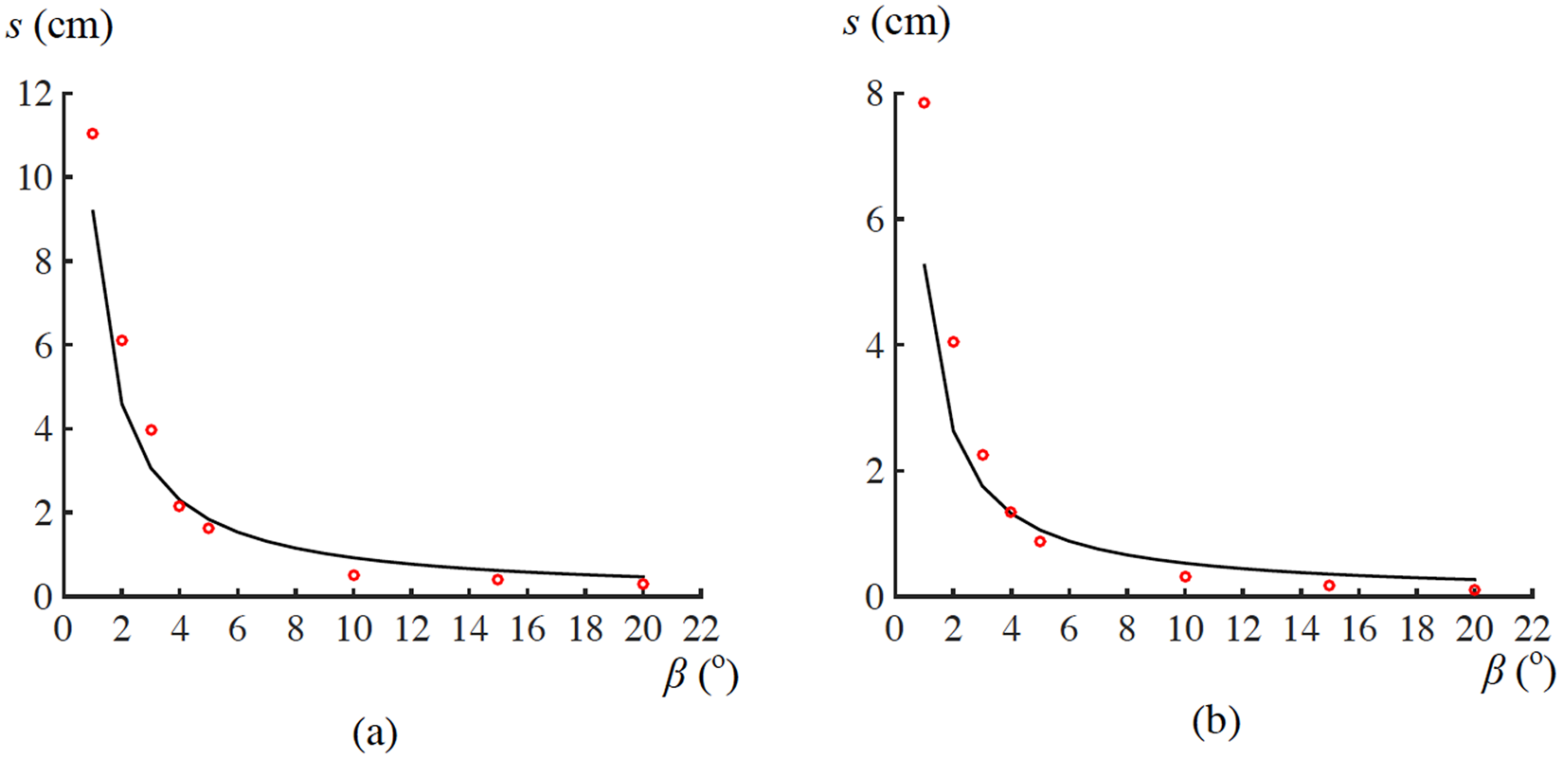
Relations between *s* and *β* for (**a**) water and (**b**) IPA drops. Scattered points represent experimental results. Lines denote theoretical predictions obtained using Eq. (16), with the values of *k* to be 0.54 and 0.64, respectively, for water and IPA. These *k* values were determined via curve-fitting of Eq. (15) in Fig. 5.

In the third experiment, the values of *t* were fixed to 2.3 ± 0.3 and 2.0 ± 0.5 mm, respectively, for water and IPA. Meanwhile, *β* was varied from 0° to 90°. When *β* ≥ 10°, due to gravity, a just released drop first slightly moved back from the pre-set location towards the closed end of a channel, and then got ejected. The errors in *t* were mainly induced by this slight movement. A set of data points (*β*, *s*) was determined. As observed from experimental results (Fig. 6), travel distances of both water and IPA drops decreased dramatically with the increase in tilt angle. For example, at *β* = 1°, they were capable of travelling 11 and 8 cm, respectively, before they finally stopped and started moving back towards the closed end of the channel. However, when tilt angles were larger than 4° and 3° for water and IPA, respectively, the corresponding travel distances were less than 2 cm. For such tilt angles, the ejection effect of a non-parallel structure may be neglected, since the travel distance is comparable to the longitudinal size of a drop.

With the aid of Eq. (15), Relation (13) may be re-written as

16 $$s=\frac{2{k}^{2}{\lambda }^{2}}{t\,\sin \,\beta }.$$

Experimental results were also compared with the values predicted using this equation. Two points were observed from this comparison for both water and IPA (Fig. 6). First, at *β* = 1°, there were some differences between theoretically predicted and experimentally measured travel distances. For water and IPA, these differences were about 2.0 and 2.5 cm, respectively. Second, these differences were less than 0.5 cm at other tilt angles. Accordingly, except for *β* = 1°, experimental results approximately matched theoretical predictions.

During the process of heating a drop, temperature distribution on the substrate may no be uniform. Accordingly, in the fourth experiment, we examined the effect of temperature gradient on a Leidenfrost drop. As detailed in supplementary information, the corresponding testing results indicate that, in our experiments, temperature variation of the substrate had negligible effect on the moving direction of a Leidenfrost drop.

Surface tension of a liquid decreases with the increase in the temperature. Also, surface tension difference between two opposite edges of a liquid drop or a solid fragment may induce a directional movement of the drop^40^ or fragment^41,42^. In Leidenfrost state, the entire bottom surface of a drop reaches its boiling point. As such, it is expected that there is negligible temperature variation across the bottom surface. Consequently, there should be little difference between surface tensions at two opposite edges of a drop. Thus, in our tests, although there existed a temperature variation on the substrate, this variation did not entrain a Leidenfrost drop.

## Applications

**Figure 7:**
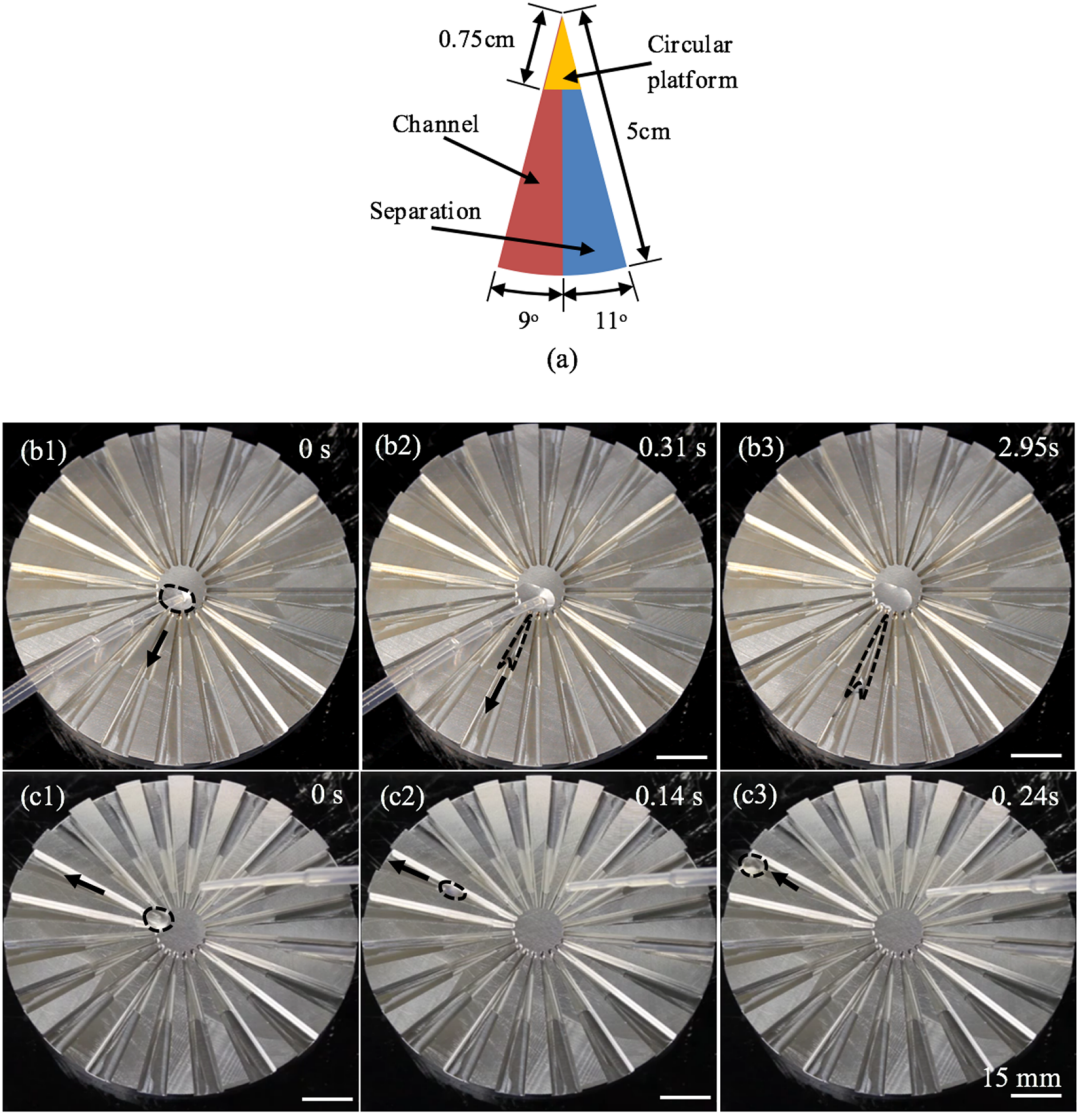
(**a**) Sketch of a unit cell of a repeller. (b1)–(b3) A water drop spread in a channel of the repeller at room temperature. (c1)–(c3) A water drop ran inside a channel on the repeller with an average speed of 15.7 cm/s at 250 °C. (**a**) is top view, while (b1)–(c3) are perspective views.

**Figure 8:**
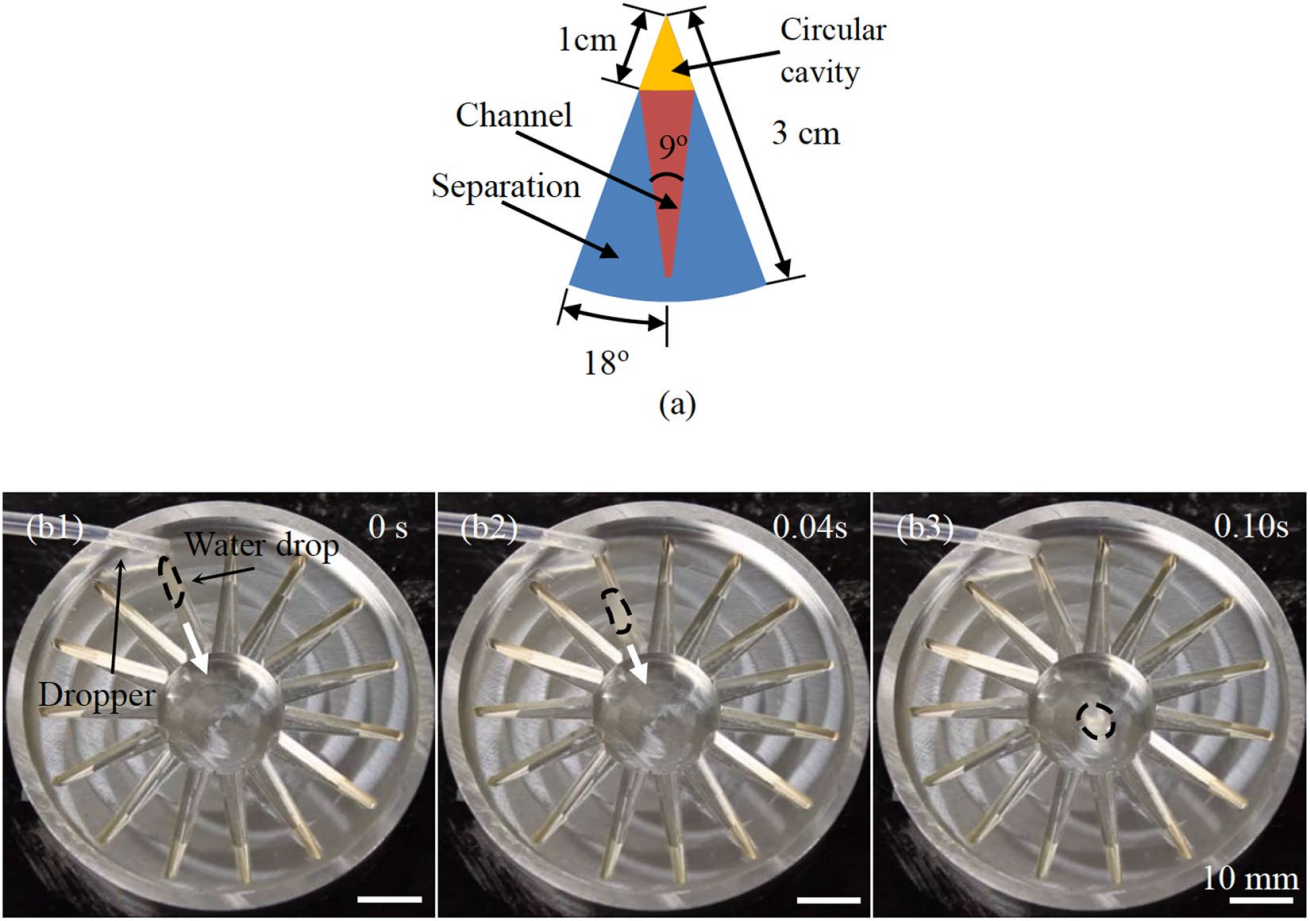
(**a**) Sketch of a unit cell of a trap (top view). (b1)–(b3) A water drop moved from the perimeter to the central cavity of the trap along a channel with an average speed of 13.9 cm/s at 250 °C (perspective views).

**Figure 9:**
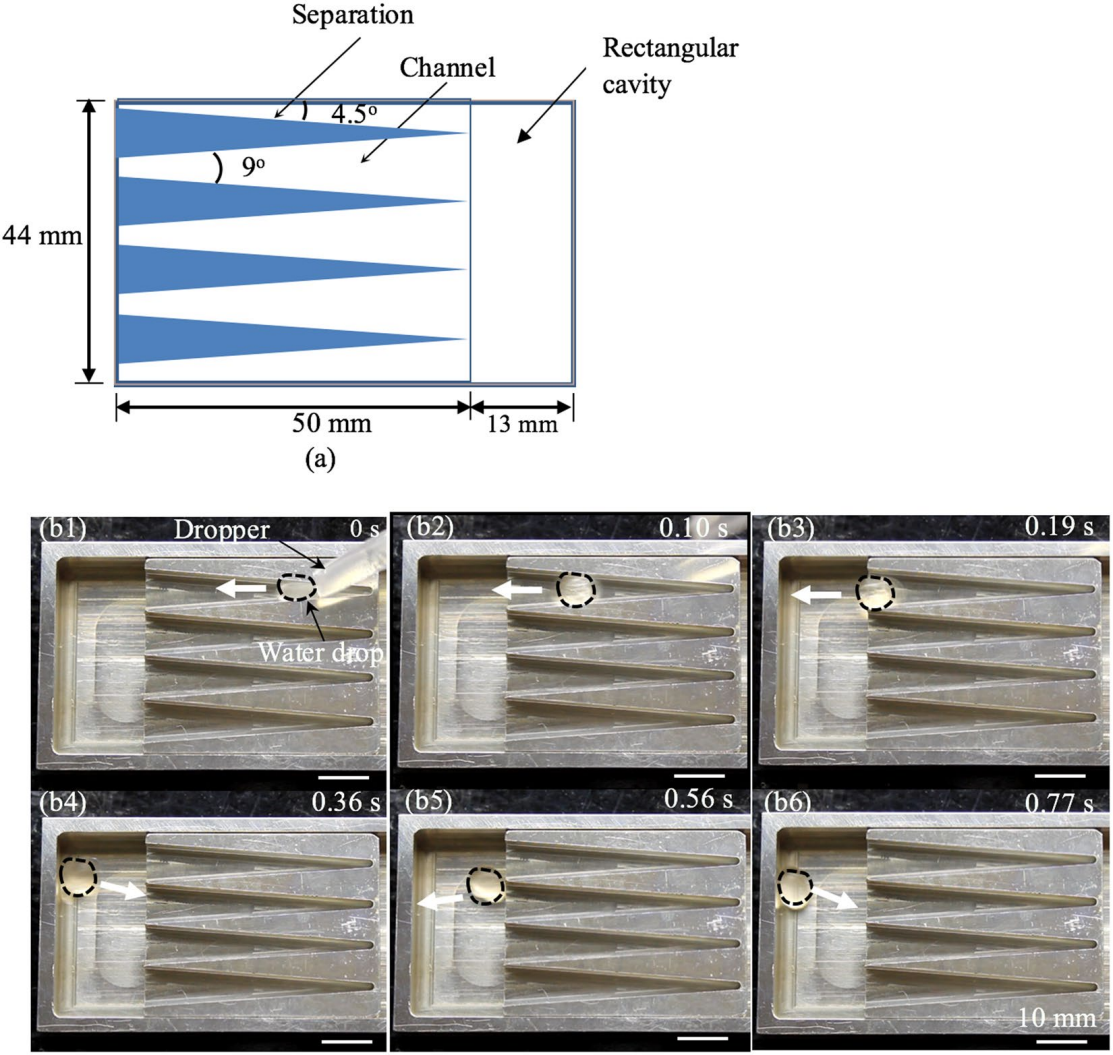
(**a**) Sketch of a guide. (b1)–(b3) A water drop ran inside a channel of the guide with an average speed of 17.4 cm/s at 250 °C. (b4)–(b6) First oscillation of the drop in between the two opposite sidewalls of the rectangular cavity with an average speed of 0.4 cm/s, and the oscillation died after five cycles. (**a**) Is top view, while (b1)–(b6) are perspective views.

According to theoretical and experimental results that we obtained in the case of the channel, three devices have been further developed to control the motions of Leidenfrost drops: repeller (Fig. 7 and Supplementary video 1), trap (Fig. 8 and Supplementary video 2), and guide (Fig. 9 and Supplementary video 3). Representative results on water drops are given here, while those for IPA are not shown. The three devices were all manufactured using a computer numerical control machine. Each device includes an array of channels. Similar to the previously tested channel, every channel in a device is also 4 mm deep.

The design of the repeller is shown in Fig. 7(a). The repeller includes a circular Al disk with a diameter of 5 cm (Fig. 7a). An array of concentric channels is incorporated on the disk. Each channel has two non-parallel sidewalls, which form an apex angle of 9°. The channel separation has a wedge shape with an apex angle of 11°. A prototype has been fabricated and tested (Fig. 7b and c). When water and IPA drops were placed on the disk at room temperature, they just spread inside channels (Fig. 7b). However, in Leidenfrost states, these drops quickly moved away from the disk center along the channels till they ran out of the disk. It took no more than 0.5 s for a drop to run through a channel. Representative movement of a single water drop was given in Fig. 7(c), while those of multiple drops were presented in Supplementary video 1. The water drops did not have identical sizes. Also, they were not placed exactly at the same location of the repeller. Accordingly, their speeds were not the same, and varied in the range of 15.1–17.2 cm/s. For IPA, the speeds ranged from 8.2 to 9.9 cm/s. These testing results indicate that the repeller is efficient in quickly removing Leidenfrost drops from the disk area.

In addition, a trap was designed and fabricated. It was a circular Al plate with an array of inward channels and a 8-mm-deep central cavity (Fig. 8). As in the cases of ratchet trap^43^ and bowl structure^44^, Leidenfrost drops on the perimeters of the plate have been successfully guided to the central cavity through the inward channels (Fig. 8 and Supplementary Video 2). On this device, water and IPA drops had the speeds of 10.0–21.6 cm/s and 7.0–13.6 cm/s, respectively. The moving direction of the drops on the trap was opposite to that in the case of the repeller.

Furthermore, a guide was developed (Fig. 9a). It included an array of parallel channels. The guide directed Leidenfrost drops to move along the same direction. The water and IPA had the speeds of 15.8–19.2 cm/s and 9.5–14.0 cm/s, respectively (Fig. 9b and Supplementary video 3). The ejected drops were collected in an 8-mm-deep rectangular cavity, which was connected with the channels. As these drops collided with the cavity sidewalls, due to loss of kinetic energy during the collision, their speeds were much reduced. After a few cycles of oscillation inside the cavity, an ejected drop stopped its movement.

The testing results on the three devices, together with those of the second and third experiments, have validated two of the theoretical predictions: i) once Leidenfrost drops contact two non-parallel sidewalls, they move towards the diverging end of the corresponding structure; and ii) these drops have ejection speeds with an order of 10 cm/s when a non-parallel structure has a mm-scaled gap. In addition, in comparison with ratchets, the non-parallel structures have relatively simple geometry, making it easier to incorporate them into a solid surface for handling Leidenfrost drops. Also, they have a better control of moving directions, since the drops transport inside their confined geometry. On the other hand, a ring of ratchets enables a Leidenfrost drop to have a circular motion^45^, while it may be difficult to do so using a non-parallel structure.

## Summary and Conclusions

In this work, we explored the behavior of a Leidenfrost drop between two non-parallel sidewalls of a structure through theoretical and experimental investigations. According to the derived theoretical model, once a Leidenfrost drop has contact with the two sidewalls of the structure, it is capable of self-transporting towards the diverging direction of the structure on a horizontal plane. Furthermore, when a substrate is tilted by no more than 4° and 3°, respectively, for water and IPA, an ejected Leidenfrost drop may still travel a cm-scaled distance. On the basis of this understanding, a group of devices have been developed: repeller, trap, and guide. The repeller was capable of removing these drops from a solid surface, the trap was suited for transporting drops from different locations to its center, and the guide was able to make the drop have a unidirectional movement. On these devices, Leidenfrost drops of water and IPA had speeds with an order of 10 cm/s, which was in the same order as their counterparts on ratchets. The three devices provide new tools for controlling the movements of Leidenfrost drops.

## Electronic supplementary material

Supplementary Information

Video 1

Video 2

Video 3
