# Supplementary material for: Self-propulsion of Leidenfrost Drops between Non-Parallel Structures
Source: Sci Rep. 2017 Sep 20;7:12018. doi: 10.1038/s41598-017-12279-6 (PMC5607289; doi:10.1038/s41598-017-12279-6)
Supplement: Supplementary file 1 — Supplementary Information [file 41598_2017_12279_MOESM1_ESM.pdf]

Supplementary information

Self-propulsion of Leidenfrost Drops between Non-Parallel Structures

*Cheng Luo, Manjarik Mrinal, and Xiang Wang

Department of Mechanical and Aerospace Engineering, University of Texas at Arlington

500 West First Street, Woolf Hall 226, Arlington, TX 76019, the United States of America;

*email: chengluo@uta.edu

This supplementary information includes two parts: details of the fourth experiment, and three videos.

I. Details of the fourth experiment

A hot plate has a circular heating zone in its center. Outside this zone, temperature is much lower. We put one end of a 16-cm-long Al plate on the center of the heating zone, while the other end outside this zone. In our tests, when the hotter end of the plate was heated to a temperature between 330 and 360 ^{o}C, the colder one had a temperature in the range of 250 to 275 ^{o}C. Accordingly, every point of the plate had a temperature above Leidenfrost points of both water and IPA. Meanwhile, in these tests, the temperature difference between the two ends of the plate created a gradient in the range of 5.0 to 7.5 ^{o}C /cm from the hotter to the colder end. This temperature gradient was much larger than the maximum possible one of 2 ^{o}C/cm in our first three experiments. When either a water or IPA drop was put in the center of the plate, we did not observe that the corresponding drop moved along a particular direction. However, when either end of the plate was raised up to form a tilt angle of 1°, this drop always ran downhill. In contrast, when tilt angle was 1°, water and IPA drops, which were ejected from a channel, could still travel uphill by 11 and 8 cm, respectively (Fig. 6). In addition, we also specifically examined

the effect of temperature gradient on IPA, when temperature ranges of the two ends of the plate were 197-215 ^{o}C and 135-145 ^{o}C, respectively. The average temperature gradient was 4.1 ^{o}C/cm. In the corresponding tests, we did not observe any obvious effect of temperature gradient as well.

II. Three videos

Video 1: Movements of multiple water drops on a repeller at 250 ^{o}C, which corresponds to Fig. 7.

Video 2: Movements of multiple water drops on a trap at 250 ^{o}C, which corresponds to Fig. 8.

Video 3: Movements of multiple water drops on a guide at 250 ^{o}C, which corresponds to Fig. 9.
